# Paper-Based Substrate for a Surface-Enhanced Raman Spectroscopy Biosensing Platform—A Silver/Chitosan Nanocomposite Approach

**DOI:** 10.3390/bios12050266

**Published:** 2022-04-22

**Authors:** Yuri Kang, Hyeok Jung Kim, Sung Hoon Lee, Hyeran Noh

**Affiliations:** 1 Department of Optometry, Seoul National University of Science and Technology, 232 Gongneung-ro, Nowon-gu, Seoul 01811, Korea; eurikang@seoultech.ac.kr (Y.K.); hjkim@seoultech.ac.kr (H.J.K.); 2 Corning Technology Center Korea, Corning Precision Materials Co., Ltd., 212 Tangjeong-ro, Asan 31454, Korea; 3 Convergence Institute of Biomedical Engineering and Biomaterials, Seoul National University of Science and Technology, 232 Gongneung-ro, Nowon-gu, Seoul 01811, Korea

**Keywords:** cellulose paper, chitosan, layer-by-layer, self-assembly, nanocomposite, SERS spectroscopy

## Abstract

Paper is a popular platform material in all areas of sensor research due to its porosity, large surface area, and biodegradability, to name but a few. Many paper-based nanocomposites have been reported in the last decade as novel substrates for surface-enhanced Raman spectroscopy (SERS). However, there are still limiting factors, like the low density of hot spots or loss of wettability. Herein, we designed a process to fabricate a silver–chitosan nanocomposite layer on paper celluloses by a layer-by-layer method and pH-triggered chitosan assembly. Under microscopic observation, the resulting material showed a nanoporous structure, and silver nanoparticles were anchored evenly over the nanocomposite layer. In SERS measurement, the detection limit of 4-aminothiophenol was 5.13 ppb. Furthermore, its mechanical property and a strategy toward further biosensing approaches were investigated.

## 1. Introduction

Surface-enhanced Raman spectroscopy (SERS) is a sensitive analytical tool for the detection of chemical and biological analytes [1,2]. It reveals the intrinsic vibrational mode of chemicals in the manner of label-free detection with high sensitivity [3,4,5]. In recent years, diverse nanoscale structures, including those of noble metal nanocomposites, have led to the development of new substrates for label-free detection via Raman spectroscopy. Resonance-enhancing spaces known as “hot spots” can be generated between nanoparticles or at the sharp edges of individual nanoparticles, which locally enhances electromagnetic fields; thus, high density is regarded as the key to obtaining better Raman signals.

Since the first SERS effect was utilized in molecular detection by Richard P. Van Duyne and other peers, there have been attempts to connect it to the biosensing approach using biosensing molecules like enzymes and antibodies [6,7]. SERS benefits from multiplex detection and spectrometric approaches in biosensing. Also, the feasible formation of coordinate bonds between those sensing molecules and noble nanoparticles makes SERS highly applicable to biosensor research, i.e., thiol–gold, thiol–silver, amine–gold, and so on. Combined with established biosensing strategies such as ELISA [8], it can expand the analytical performance of biosensing to ultrasensitive levels. There have been several reports showing how SERS can be applied to biosensing [9,10,11].

SERS-active substrates are generally categorized as colloidal liquids or solid materials. Colloidal SERS substrates are relatively simpler in their preparation, and they can derive various shapes of nanoparticles to target a specific analyte. However, colloidal stability is more important due to the risk of particle aggregation, precipitation, and loss of colloidality. Thus, solid materials binding to individual nanoparticles, such as paper, glass, silicone, polydimethylsiloxane (PDMS), graphene nanosheets, and polymer films, may be preferred in some applications [12,13,14,15,16]. In particular, porosity is widely sought as it effectively improves the density, dispersion, and consistency of hot spots over a whole dimension [5,17,18,19]. Moreover, the following features of these materials are thought to be beneficial in SERS analysis: (1) effective adsorption of analytes, (2) ease of multiple adjacent nanostructure formation to increase hot spots, (3) support fixtures for nanoparticle growth, and (4) filtering for selective detection. Some recent studies in the literature elucidated how such nanoporous materials improve analytical performance. A network of carbon wires and pores showed high absorptivity and high-density hot spots, enhancing signals [20]. A porous silicon material resulted in signal enhancement around 5 times stronger than that of SERS using a flat silicon surface [21]. A nanoparticle-embedded polymer hydrogel showed high sensitivity and filtration functions excluding small matrix molecules [22].

Recently, paper as a porous base material has received attention due to its reformability, low cost, and biocompatibility, to name but a few [23,24,25,26]. There are some notable reports exploiting its filtrating and microfluidic characteristics for renovative SERS sensing [23,27,28,29]. These paper-based substrates can be fabricated by well-established technologies, including core–shell nanoparticles/hybridization of metals [30], chemical vaporizing deposition [31,32], and hydrophobization with wax or siloxane [24], all of which enhance sensitivity in SERS analysis.

In this study, we maximize the porosity of paper using nanoporous silver/chitosan nanocomposites on the cellulose surface, which increase the number of hot spots and provide a filtration function for small molecules. Chitosan was chosen due to its chemical similarity to cellulose. That is, the whole structure can be stabilized in a paper matrix, enhancing its mechanical and thermal stability to a level suitable for SERS-active paper substrates [33,34]. Layer-by-layer (LbL) processing was employed for a better firm structure of this soft material. This low-cost silver/chitosan nanocomposite substrate was used to detect a low concentration of a model small chemical, 4-aminothiophenol (4-ATP). Lastly, we suggest a way to enhance the analytical performance of this substrate by simply cutting it.

## 2. Materials and Methods

### 2.1. Materials and Reagents

Chitosan oligosaccharide lactate ((C_12_H_24_N_2_O_9_)_n_, >90% deacetylated, average M_n_ = 5000, oligosaccharide 60%), acetic acid (CH_3_COOH, 99%), and 4-aminothiophenol (C_6_H_7_NO, >97%) were purchased from Sigma Aldrich. Silver nitrate (AgNO_3_, >99%), sodium citrate dihydrate (Na_3_C_6_H_5_O_7_·2H_2_O, >99%), and anhydrous absolute ethanol (C_2_H_5_OH, 99.5%) were purchased from Daejung Chemical, Inc. (Siheung-si, Gyeonggi-do, Korea), and sodium hydroxide (NaOH, >93%) and sodium borohydride (NaBH_4_, >98%) were purchased from Duksan Chemical, Inc. (Ansan-si, Gyeonggi-do, Korea). Whatman standard chromatography paper (Whatman 1 CHR, Cytiva, Marlborough, MA, USA) and a dialysis tubing cellulose membrane (flat width 25 mm, Sigma Aldrich, Burlington, MA, USA) were used as the base materials in SERS measurement and SEM imaging, respectively.

### 2.2. Fabrication of the Paper SERS Substrate

**Figure 1:**
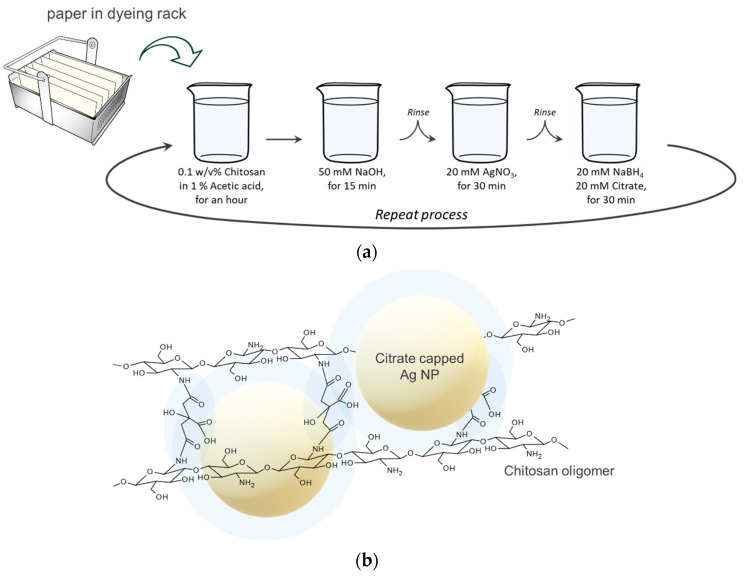
(**a**) Schematic illustration of the layer-by-layer process used to coat cellulose paper with silver/chitosan nanocomposite, where the paper substrate was submerged in each reagent solution, stepwise; (**b**) Configured silver/chitosan nanocomposite by coordinate bonds between citrate-capped silver nanoparticles and self-assembled chitosan.

Whatman standard chromatography papers 2.5 × 7.0 cm^2^ in size were employed. A slide rack was used to fabricate multiple sheets at once, prepared with slide glasses as dividers between the papers. To fabricate the silver/chitosan nanocomposite, a 0.1 *w*/*v*% chitosan solution (in 1% of acetic acid), a 50 mM sodium hydroxide solution, a 20 mM silver nitrate solution, and reductant solutions (20 mM sodium borohydride, 20 mM sodium citrate dihydrate) were prepared. The silver/chitosan nanocomposite was formed after the paper cellulose was dipped into the chitosan solution for 1 hour, the sodium hydroxide solution for 15 min, the silver nitrate solution for 30 min, and the reductant solution for 30 min, sequentially. After each immersion step, except for that in the chitosan solution, the paper was rinsed in water under magnetic stirring for 20 min. We then dried the paper in an oven at 60 degrees for 30 min, making this the LbL 1 cycle. This was repeatedly performed on the single-cycled paper via the same method described above to obtain 2 cycles, 3 cycles, and so on (Figure 1a).

To explain the synthesis process in detail, an acetic acid solution was used to dissolve the chitosan. This makes the chitosan molecules electrically positive through the protonation of amino groups at low pH, and this property increases the water solubility. The dissolved chitosan has an electrostatic attraction with cellulose, which is a polyanion, in aqueous solution. When the paper is placed in the next sodium hydroxide solution, the amine groups of chitosan become insoluble at high pH and may become harder on the paper. Rinsing off unwanted ions that are not used for binding and immersing the paper in a silver nitrate solution and a reductant solution results in negatively charged silver nanoparticles capped with a citrate capping agent. By repeating the cycle, citrate-capped silver nanoparticles can be coordinated with chitosan to obtain a three-dimensional nanocomposite (Figure 1b).

The synthesis method for the non-LbL model was identical to the first cycle step, but from the second cycle onwards, it consisted only of the silver nitrate solution and the reductant solution without the chitosan solution or the sodium hydroxide solution. The volume of solution used in the experiment was adjusted according to the size of the paper to be produced. For the ten sheets of paper mentioned above, amounts of 0.5 L of the solutions were used. After completion of production, the dried paper samples were wrapped in aluminum foil and stored under refrigeration at about 4 °C.

### 2.3. Sampling for the Detection of 4-ATP

Paper samples for the SERS measurements were cut into 4 mm diameter circles using a perforated punch. These were immersed in a 1.5 mL tube with 0.5 mL 4-ATP (12.5 ppm in ethanol) for 18 h at 45 degrees and then dried at room temperature for use with the SERS measurements. The other paper sample used here with capillary action was 4 mm wide and 20 mm long, and the tip was prepared as an isosceles triangle with a height of 4 mm. These samples were immersed in the same manner as the circle sample above, except that the volume of 4-ATP used was 1.5 mL. After immersion for 18 h, the dried sample was placed vertically in a 60 mm diameter Petri dish covered with ethanol to approximately 1 mm high. One minute after the ethanol reached the tip of the paper, it was taken out and dried at room temperature, after which a 4 mm high triangle was cut, and SERS measurements were taken.

### 2.4. Calculation of the Limit of Detection

The detection limit was obtained by the following formula:

(1) $$I_{m}=I_{bl}+k\;\sigma_{bl}$$

where *I_m_* is the minimum distinguishable intensity of the signal, *I_bl_* is the Raman signal generated by a blank measurement of the SERS substrate in the absence of the analyte. *k* is the proportionality constant, and *σ_bl_* is the standard deviation of blank measurements [35]. In addition, the amount of analyte providing 3 times the *σ_bl_* value as a signal equal to or greater than the signal of the blank was considered (using *k* = 3 proposed by Kaiser [36]).

(2) $$I=m\;C_{m}+I_{bl}$$

*C_m_* was calculated by substituting *I_m_* obtained by Equation (1) into *I* of the quantified linear function in Equation (2). *m* is the slope of the calibration curve at the concentration of interest, and *C_m_* is the concentration at the limit of detection.

### 2.5. Instruments

Optical microscopy (Zeiss Primo star, ZEISS International, Jena, Germany) was used to obtain the color shift of the LbL model compared to the non-LbL model. Field emission scanning electron microscopy (FE-SEM, Hitachi SU8010, Hitachi, Tokyo, Japan) was used to image the surface and cross section of the substrate. The cross-sectional view was obtained using this cellulose membrane (thickness of around 20 μm) and by fracturing after immersing it in cryo liquid. The silver/chitosan nanocomposite layered samples were pre-treated with a Pt coating and then subjected to analysis. A universal testing machine (Instron 3400 series) was used for micro-tensile testing of the LbL fabricated paper samples. The Raman signals were measured using a C12710 Raman spectrometer from Hamamatsu. This device has a built-in laser with a wavelength of 785 mm. Here, the laser output was 50 mW (Raman mode) or 3 mW (SERS mode) and the acquisition time was 1500 ms. Raman intensity values were determined from 10 spots and averaged.

### 2.6. Statistics

Statistical analyses using Student’s t-test and the standard deviation (of all error bars) were conducted using SPSS 18 (SPPS, Inc. Chicago, IL, USA) and Microsoft Excel (Microsoft, Inc. Redmond, WA, USA).

## 3. Results and Discussion

### 3.1. Characterization of Silver/Chitosan Nanocomposite Layered Paper

#### 3.1.1. Physical Properties

**Figure 2:**
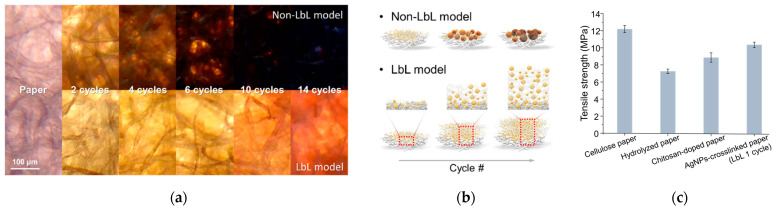
(**a**) Optical micrographs of LbL and non-LbL paper that underwent the fabrication process for 14 cycles, representing the effects of chitosan on colloidal stability; (**b**) Illustration showing how chitosan oligomer prevents the aggregation of particles during the LbL process; (**c**) Changes in the tensile strength of the substrate when proceeding with each step, implying mechanical enhancement by chitosan self-assembly and crosslinks with silver nanoparticles.

To confirm the in situ formation of silver/chitosan nanocomposites on paper, the optical properties of the LbL model and the non-LbL model were compared. The first cycle performed on cellulose paper was identical in both models, i.e., chitosan immersion followed by the reduction of the silver nanoparticles (Ag NPs). The LbL model underwent repeated production steps sequentially, while in the non-LbL model, the reduction of Ag NPs was performed without submersion in chitosan. Comparing the optical micrographs of the two models in Figure 2a, a dramatic color change in the non-LbL model was observed. The distinct decrease in the brightness of the non-LbL sample is presumably due to the interference of the optical path, judging from the increase in the size or the aggregation of Ag NPs. The paper formulated by the non-LbL model turned darker during the cycling process because the spacing of the paper fibers became narrow. When the same molar concentration of silver ions was added at each cycle in the LbL model, fiber spaces were possibly preserved by the chitosan multilayer. Since chitosan can act as a spacer between Ag NPs, the aggregation of Ag NPs is prevented by chitosan in the LbL model, as illustrated in Figure 2b [37,38]. The optical intensities in both red and green maintained less than a 20% change in RG chromaticity (Figure S1). This observation indicated that the aggregation of Ag NPs was prevented by crosslinked chitosan in the LbL model, such that nanoparticles of similar sizes could be distributed without aggregation or unwanted growth.

A micro-tensile test was conducted to evaluate the physical properties of the nano-scale material inside the nanocomposite layered paper. With the acid-catalyzed glycosidic hydrolysis, the tensile strength of the hydrolyzed paper was significantly reduced compared to that of the cellulose paper (Figure 2c). Meanwhile, the physical strength of the substrate was recovered through the doped chitosan crosslinked with Ag NPs.

#### 3.1.2. Morphology of the Silver/Chitosan Nanocomposite

**Figure 3:**
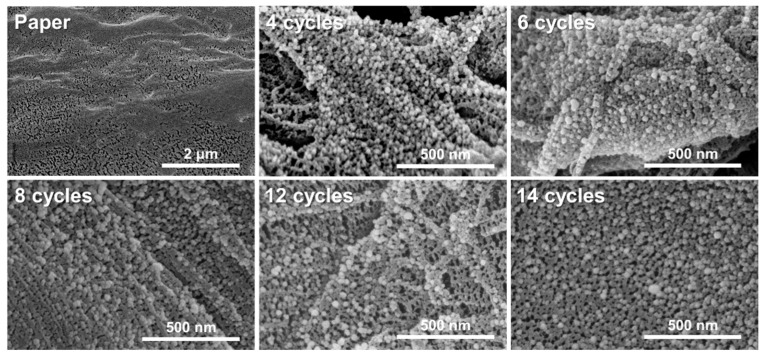
SEM micrographs of the resulting LbL papers after each cycle (from top right: original paper; 4, 6, 8, 12, and 14 cycles) where silver nanoparticles are evenly anchored to the surface of fibers without significant changes in particle size.

The distribution and conformation of the nanoparticles are two of the major sources of SERS enhancement. Accordingly, the morphologies of the silver/chitosan nanocomposites on cellulose paper were characterized by SEM. Chemically untreated chromatography paper was chosen as a control paper; however, it was easily torn by the electron beam of the SEM and was photographed at low magnification. Figure 3 shows the evenly spread Ag NPs over the surface of the cellulose fiber as a consequence of LbL cycling. The size of the Ag NPs did not increase by more than 50 nm, even when the cycling was repeated. The histogram distribution also confirmed that the Ag NP reduction rates were consistent throughout the cycles, exhibiting an average nanoparticle size of 26.99 ± 5.74 nm (Figure S2). We believe that the particle size is not affected by further solution immersion steps as the citrate works as capping agent, stabilizing and inhibiting the over-growth of Ag NPs while also preventing their aggregation and coagulation formed in the previous cycle. Sodium citrate can also chemically crosslink with chitosan between protonated amines and carboxylate ions through a heat treatment [39,40]. Since the paper after the dipping step in the citrate-containing reductant goes through an oven-drying process at about 60 degrees, the above reaction is likely to occur. Chitosan not only binds to silver nanoparticles but also binds to the citrate capping agent to form a stable three-dimensional layer. Therefore, it appears that the silver ions are prevented from diffusing and, consequently, have a constant reduction rate and maintain a uniform particle size over multiple cycles.

This corresponds to the overall Ag NP sizes in each cycle, 20 to 30 nm, as calculated from the SEM images, implying that the Ag NPs created by the LbL cycling did not aggregate but maintained a relatively constant size. It is well known that constant size and regularity of plasmonic nanoparticles are crucial factors related to SERS performance outcomes because these factors can affect the formation of local electromagnetic fields on the surfaces of the nanoparticles [41]. In the LbL model, Ag NPs between the chitosan multilayers not only prevented aggregation but also maintained a suitable size of the nanoparticles for the SERS signal.

**Figure 4:**
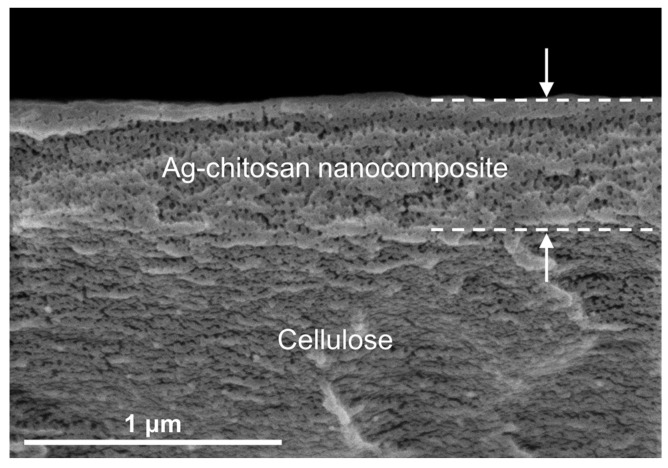
Cross-sectional view of Ag–chitosan nanocomposite on the cellulose substrate (thickness of Ag–chitosan nanocomposite, approx. 575 nm; diameter of nanopores, up to 30 nm).

Figure 4 presents an SEM image of a cross section of the nanocomposite layer on the cellulose surface (the specifications of the materials are shown in the methodology section). A cross-sectional view demonstrates the fabricated multilayer (thickness of approx. 575 nm at 14 cycles) and its nanoporous structure. Notably, the nanoporous structure was well refined and evenly formed over the layers with similar pore sizes of up to 30 nm in diameter (Figure S3). Chitosan oligomers associated with the surface shrank to themselves, while the high concentration of hydroxide induced self-assembly of these oligomers, and the structure was then fixed after being dried out. This nanoporous structure brings more functions to our substrate; it can promote the high acquisition of SERS signals as a ‘spacer’, but it can also selectively filtrate for target analytes by molecular size to allow for low matrix effects due to proteins in biological samples.

#### 3.1.3. Raman Spectroscopy

**Figure 5:**
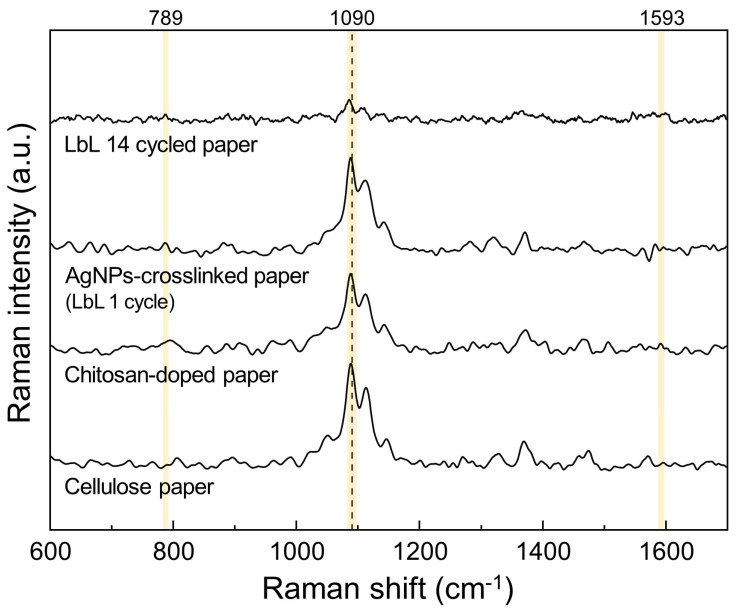
Raman spectra obtained from original cellulose, chitosan-doped, LbL 1-cycle, and LbL 14-cycle substrates, where the yellow bands represent the chemical motifs of the following: glycosidic ring stretching, 1090 cm^−1^; amine group in chitosan, 1593 cm^−1^; and silver, 789 cm^−1^.

The chemical structures of each paper layer up to 14 cycles were analyzed by Raman spectroscopy using a 785 nm incident laser at 50 mW. Figure 5 shows the Raman spectra of the untreated cellulose paper, the chitosan-doped cellulose paper, the Ag NP-crosslinked paper, and the LbL 14-cycle paper. The high-intensity peak at 1090 cm^−1^ represents C-O ring stretching and/or glycosidic ring stretching [42,43]. Bands from ring stretches were detected in all samples. Chitosan and cellulose have similar molecular structures, with the difference being the existence of an amine group, NH_2_ or NHCOCH_3_, located at the C2 site in the case of chitosan. The Raman band of the amine group in chitosan was located at 1593 cm^−1^ [44], and the silver peak was measured in the LbL 1-cycle sample at 789 cm^−1^. Comparing the Raman signals of the nanocomposite layered paper and the untreated cellulose paper, the position of the cellulose peak in the LbL 14-cycle paper was 1085 cm^−1^, which shifted to a wavenumber lower than that of the control paper. Studies of deformation mechanisms have shown that a shift in the Raman band is indicative of stress in the fiber [45,46]. The Raman shift to a lower wavenumber means that the vibrational frequency of the molecule is decreased, which could be considered as evidence of the lengthening of the chemical bond due to tensile stress. As the silver/chitosan nanocomposites were repeatedly deposited, stress was applied to the C-O stretching motion of cellulose, which is almost parallel to the chain axis, and a shift of the Raman band was observed [47].

### 3.2. SERS Measurement

#### 3.2.1. Detection of 4-Aminothiophenol

**Figure 6:**
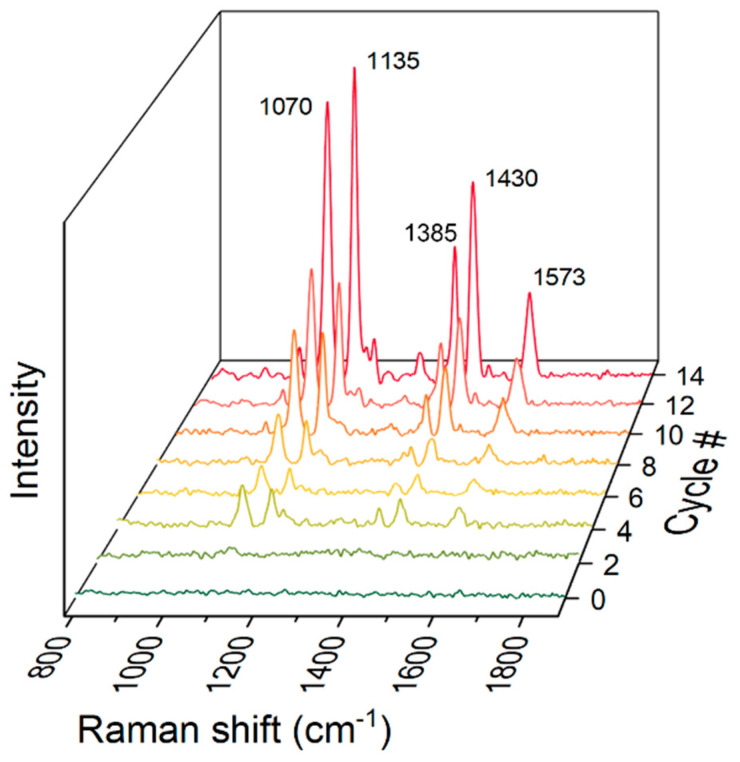
SERS spectra showing an increment in each peak of 4-ATP (12.5 ppm) with increasing number of LbL cycles.

Figure 6 shows the SERS intensity with increasing number of synthesis cycles. The standard 4-aminothiophenol (4-ATP) was employed to study the SERS performance of the silver/chitosan nanocomposite layered paper. The characteristic peak of 4-ATP is at 1070 cm^−1^, corresponding to C-S stretching vibration [48]. Each cycled LbL paper showed amplification of the Raman signal with increasing cycle number as compared to the untreated paper. It appears that the silver nanoparticles inside the nanocomposite were spaced by chitosan, effectively increasing the number of hot spots. A strong electromagnetic field occurred with the interparticle distance decreased to a few nanometers; the chitosan backbone structure prevented the silver nanoparticles from aggregating, while still keeping them quite close. In addition, the three-dimensional structure of cellulose paper and chitosan appears to have contributed to the increased hot spot density due to the porous structures of these components.

**Figure 7:**
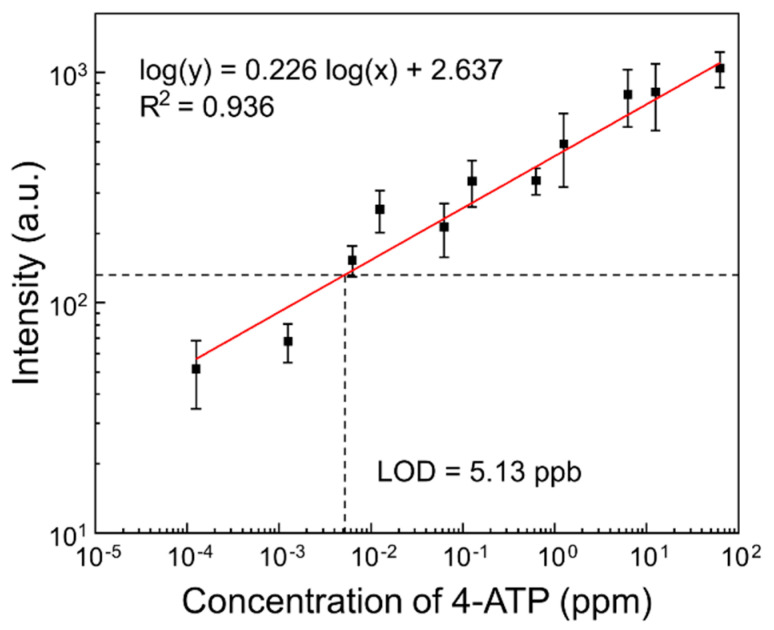
SERS intensity at 1070 cm^−1^ obtained from a series of different concentrations of 4-ATP dropped onto prepared SERS substrates (analyte concentration ranging from 0.00125 ppm to 12.5 ppm).

For quantitative analysis, a series of solutions with different concentrations of 4-ATP were measured via SERS, as shown in Figure 7. The intensity of the Raman signal at 1070 cm^−1^ increased with a rise in the concentration of 4-ATP. The bands at 1135, 1385, and 1430 cm^−1^ indicate b_2_ vibration modes that are selectively enhanced via the charge transfer mechanism, so their quantification is unstable [49,50]. Among several bands, 1070 cm^−1^, representing C-S stretching vibration, was chosen as a specific peak for quantitative analysis. The coefficient of determination (R^2^) was determined to be 0.936 according to a linear regression analysis. The detection limit was found to be 5.13 ppb, which is equivalent to approximately 41 nM. This is comparable sensitivity to that of other rapid-testing SERS substrates and very refined nanomaterials achieving nanomolar detection of environmental pollutants, while ours retains feasibility in terms of the fabrication and wettability of the paper material. We look forward to the use of this substrate as a superb candidate platform for on-site biosensors [25,51].

#### 3.2.2. Application to Biosensing Approaches

While our SERS substrate has shown competent functionality, further potential was observed when recalling the traits of paper celluloses: large surface area, conformation of the scaffold structure, capillary flow, and free evaporation, to name but a few. This implies that this substrate can be used to implement novel biosensing applications, for instance, the real-time monitoring of bacterial metabolites during cultivation [52]. The nanoporous structure observed via SEM is thought to filter large matrix molecules like proteins, increase the surface area, and specifically target small molecules. Further, capillary flow and free evaporation have been considered a simple but effective strategy to concentrate analytes.

**Figure 8:**
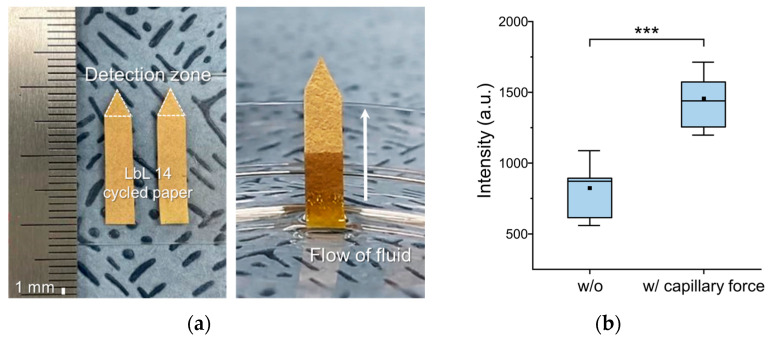
(**a**) Photograph of LbL paper substrate cut into an arrow shape with a lateral transportation and detection zone where the sample liquid moves and is vaporized, consequently focusing analyte molecules on the tip; (**b**) Signal enhancement by simply cutting the LbL paper substrate (black dot, mean; black solid line in box, median; box height, sample variability from 25% to 75%; error bar, standard deviation; *** *p* < 0.001).

In paper, the cellulose fibers are irregularly interspersed, and it is expected that the spaces inside show microtubule-like behavior; thus, we tested the efficiency of analyte-containing fluid transport in the paper. After the chromatography process was carried out with ethanol as a solvent, an isosceles triangle with a height of 4 mm was cut out and used for 10 SERS measurements from different spots (Figure 8a). The Raman signals of the control and the experimental sample with 12.5 ppm of 4-ATP at 1070 cm^−1^ were 823.39 and 1455.02, respectively, showing an improvement by approximately 76% (*p* = 0.0006, *p* < 0.001; differences for which *p* was less than 0.01 were regarded as statistically significant), as shown in Figure 8b. It was noted that the solvent migrated together with the analyte, contributing to an increase in the molecular density of the analyte around the hot spots. Also, the relative standard deviation (%RSD) of the Raman signal was reduced from 32.1% to 17.7%. Consequently, the capillary phenomenon, enabled by the characteristics of the paper, enhanced the detection sensitivity of the paper sensor.

## 4. Conclusions

A new approach to generating high-density hot spots in a paper matrix was introduced herein. The layer-by-layer process with a pH-based self-assembly step enabled feasible and robust fabrication that resulted in good traits regarding mechanical characterization and surface-enhance Raman spectrometry. The synthesized Ag nanoparticles maintained a certain size even after repeated cycles, at an average of 26.99 ± 5.74 nm, which is suitable for amplifying the electromagnetic field and contributing to enhanced SERS signals. These findings show that the cellulose, chitosan, and silver nanoparticles were strongly bonded by coordinate bonds, indicating that coating on fibrils was successfully achieved. In SERS signal analysis using 4-ATP, sufficiently enhanced signals were obtained to detect 5.13 ppb. Meanwhile, combining the general traits of paper celluloses and the functionality of this nanocomposite offers potential for novel biosensing applications, such as real-time monitoring during microbial cultivation and lateral flow sensing, as we mentioned. Our results obtained by employing a capillary-actuated fluid transport scheme showed how this substrate can be used for applications ranging from simple analyte concentration up to a more complicated sensing approach. We expect that this multilayered paper will serve in the detection and monitoring of various disease factors in human body fluids, including blood, tears, and sweat, as well as environmental samples.

## Data Availability

The data presented in this study are available in https://figshare.com/search?q=10.6084%2Fm9.figshare.19635069, (accessed on 16 March 2022).

## Supplementary Materials

The following supporting information can be downloaded at: https://www.mdpi.com/article/10.3390/bios12050266/s1, Figure S1: RG chromaticity diagram of the LbL and non-LbL model paper samples up to 14 cycles. The number of cycles of both models increases along the direction of the arrow. Inset pictures are optical micrographs of the cellulose paper and 14 cycles in both models; Figure S2: Histogram of the diameters of the Ag NPs overall; Figure S3: Cross-sectional SEM image of an Ag–chitosan nanocomposite with pore size measurements; Figure S4: Tensile strength of silver/chitosan nanocomposite layered papers from cellulose paper (0 cycles) to 14 cycles [53].
