# Supplementary material for: Paper-Based Substrate for a Surface-Enhanced Raman Spectroscopy Biosensing Platform—A Silver/Chitosan Nanocomposite Approach
Source: Biosensors (Basel). 2022 Apr 22;12(5):266. doi: 10.3390/bios12050266 (PMC9138243; doi:10.3390/bios12050266)
Supplement: Supplementary file 1 [file biosensors-12-00266-s001.zip › biosensors-1662739-supplementary.pdf]

Supplementary material

Paper-based surface-enhanced Raman spectroscopy biosensing platform – a silver/chitosan nanocomposite approach

Yuri Kang [1], Hyeok Jung Kim [1], Sung Hoon Lee [2,*] and Hyeran Noh [1,3,*]

[1] Department of Optometry, Seoul National University of Science and Technology, 232 Gongneung-ro, Nowon-gu, Seoul, Republic of Korea; eurikang@seoultech.ac.kr (Y.K.); hjkim@seoultech.ac.kr (H.J.K.)

[2] Corning Technology Center Korea, Corning Precision Materials Co., Ltd., 212 Tangjeong-ro, Asan 31454, Chungcheongnam-do, Korea

[3] Convergence Institute of Biomedical Engineering and Biomaterials, Seoul National University of Science and Technology, 232 Gongneung-ro, Nowon-gu, Seoul, Republic of Korea

* Correspondence: sunghoonlee@corning.com (S.H.L.), hrnoh@seoultech.ac.kr; Tel.: +82 02-970-6231 (H.N)

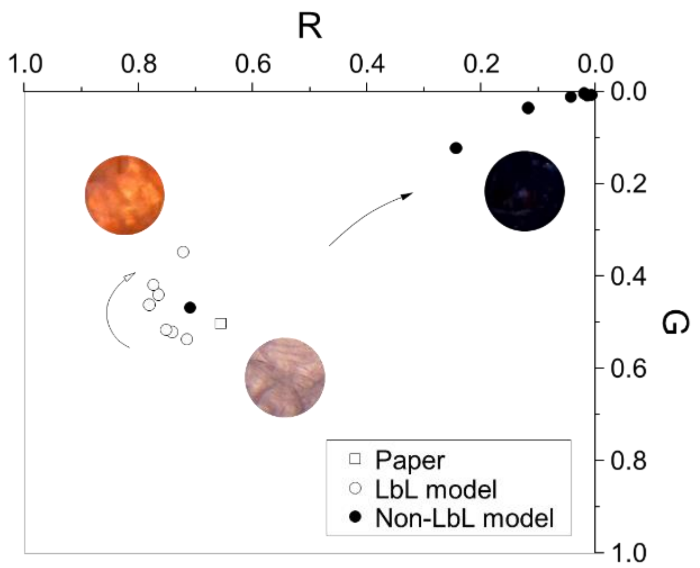

Figure S1. RG chromaticity diagram of the LbL and non-LbL model paper samples up to 14 cycles. The number of cycles of both models increases along the direction of the arrow. Inset pictures are the optical micrographs of cellulose paper and 14 cycles of both models.

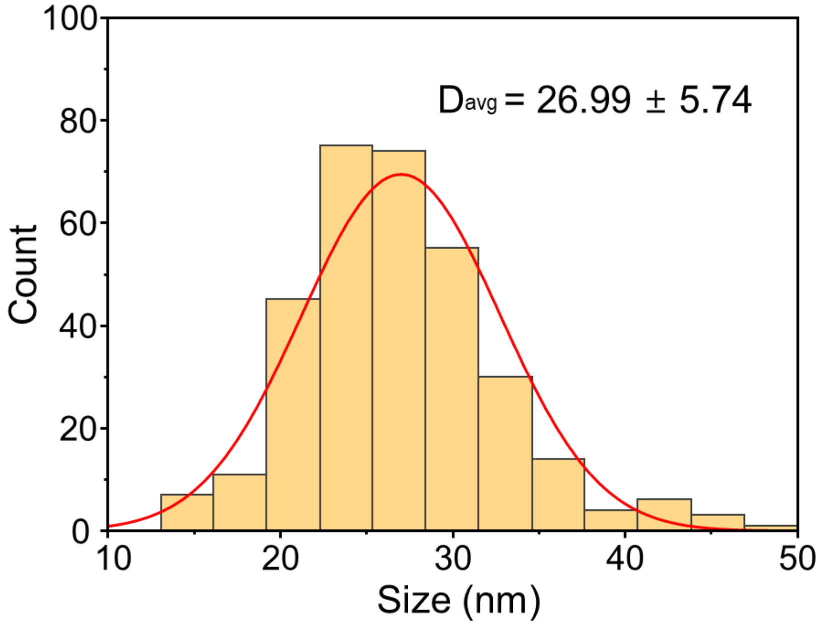

Figure S2. Histogram of the diameters of the Ag NPs overall.

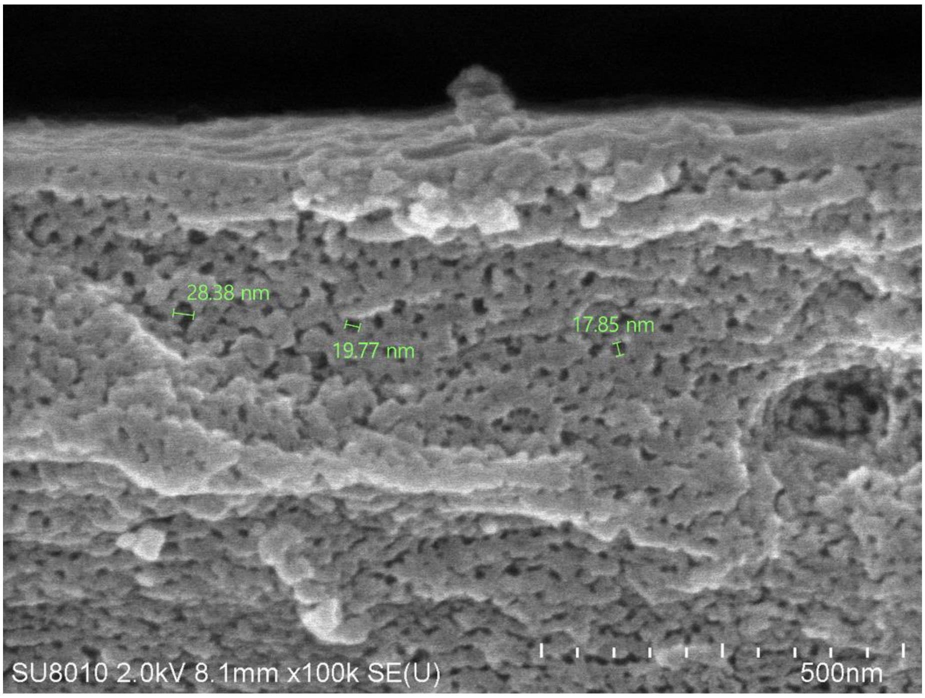

Figure S3. Cross-sectional SEM image of an Ag-chitosan nanocomposite with pore sizes measurement.

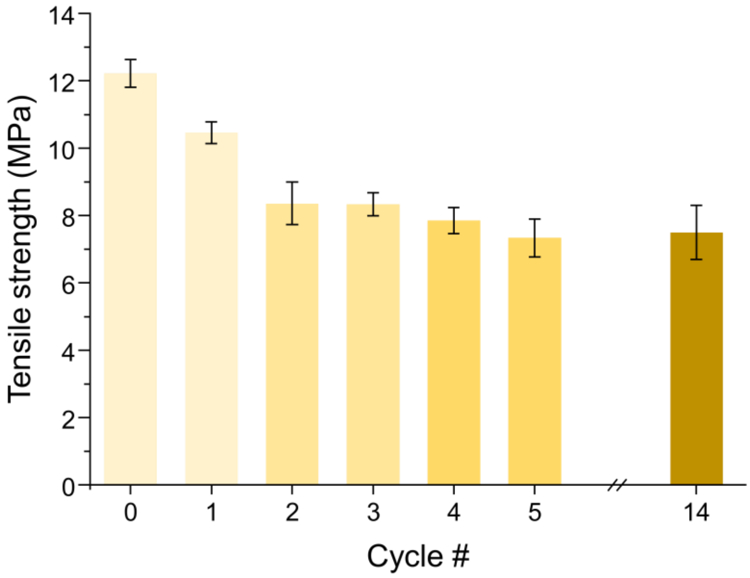

Figure S4. Tensile strength of silver/chitosan nanocomposite layered papers from cellulose paper (0 cycles) to 14 cycles.

The tensile strength of the LbL paper that had been repeatedly synthesized up to 14 cycles gradually decreased and eventually became saturated. Cellulose has a high level of crystallinity, which is reflected in its high tensile strength. However, the binding with silver/chitosan nanocomposite limited the crystallinity of the cellulose fiber, causing a decrease in the molecular-level tensile strength [1]. Also, the tensile strength is considered saturated because the spaces in the paper are limited.

Reference

1. Azevedo, E. P.; Retarekar, R.; Raghavan, M. L.; Kumar, V. Mechanical properties of cellulose: chitosan blends for potential use as a coronary artery bypass graft. Journal of Biomaterials Science, Polymer Edition 2013, 24, 239–252.
